# Aging in a Long-Lived Clonal Tree

**DOI:** 10.1371/journal.pbio.1000454

**Published:** 2010-08-17

**Authors:** Dilara Ally, Kermit Ritland, Sarah P. Otto

**Affiliations:** 1 Department of Biological Sciences, University of Idaho, Moscow, Idaho, United States of America; 2 Department of Zoology, University of British Columbia, Vancouver, British Columbia, Canada; 3 Department of Forest Sciences, University of British Columbia, Vancouver, British Columbia, Canada; University of Newcastle upon Tyne, United Kingdom

## Abstract

From bacteria to multicellular animals, most organisms exhibit declines in survivorship or reproductive performance with increasing age (“senescence”) [1],[2]. Evidence for senescence in clonal plants, however, is scant [3],[4]. During asexual growth, we expect that somatic mutations, which negatively impact sexual fitness, should accumulate and contribute to senescence, especially among long-lived clonal plants [5],[6]. We tested whether older clones of *Populus tremuloides* (trembling aspen) from natural stands in British Columbia exhibited significantly reduced reproductive performance. Coupling molecular-based estimates of clone age with male fertility data, we observed a significant decline in the average number of viable pollen grains per catkin per ramet with increasing clone age in trembling aspen. We found that mutations reduced relative male fertility in clonal aspen populations by about 5.8×10^−5^ to 1.6×10^−3^ per year, leading to an 8% reduction in the number of viable pollen grains, on average, among the clones studied. The probability that an aspen lineage ultimately goes extinct rises as its male sexual fitness declines, suggesting that even long-lived clonal organisms are vulnerable to senescence.

## Abstract 2

Using genetic estimates of clone age in trembling aspen, this study demonstrates a significant decline in male sexual fitness with increasing age, showing that long-lived clonal organisms are vulnerable to aging.

## Author Summary

Aging has been demonstrated in many animals and even in bacteria, but there is little empirical work showing that clonal plants age. Evidence for aging in long-lived perennials is scarce because it typically requires survivorship or fecundity schedules from long-term demographic data. Given the extreme lifespan of many long-lived perennials, it is difficult to follow cohorts of individual clones to collect late-life survivorship or fertility. Our work offers a novel approach for obtaining late-life demographic data on a clonal species by using genetic data to estimate the age of individual clones. We studied plant clones in a natural population of trembling aspen, which grows clonally via lateral root suckers. By coupling estimates of each clone's age with a measure of its male reproductive performance, we show that long-lived plant clones do senesce. Although clonal plants have the capacity for continued growth and reproduction even late in life, mutations that reduce fertility can accumulate because selection on sexual fitness is absent during clonal growth, potentially explaining senescence in this species.

## Introduction

Many species of animals, and even bacteria, demonstrate a decline in survivorship or reproductive performance with increasing age (“senescence”) [1],[2]. Evidence for senescence in perennial plants, however, is scant [3],[4],[7]. One feature that distinguishes plants from many animals is indeterminate growth. Indeterminate growth is particularly extreme in clonal plants, where an individual (genet/clone) can continually produce new physiological and demographic units (ramets) without undergoing sex. Senescence is thought to be a by-product of natural selection, acting most effectively early in life, when many species have the greatest reproductive value [8]. Mutations that are deleterious to late-life survival and reproduction can spread because of early-life benefits and/or because selection against them is too weak when few individuals survive to old age [9]. Because many perennial plants and especially clonal plants continue to grow throughout life, their reproductive potential can rise over time [10]. This rising reproductive potential counters the decline in natural selection that accompanies aging, allowing selection to remain effective even in late life. It is this characteristic of indeterminate growth in perennial plants that has led some to speculate that these organisms defy aging [1],[3],[10]–[12].

Indeterminate growth is, however, a double-edged sword. Although it facilitates genet growth and renewal, it also results in more mitotic cell divisions, increasing the accumulation of somatic mutations [13]. Because somatic mutations arise in the cells of the plant body (roots and/or above-ground mass), they can be passed on to subsequent ramet generations. Furthermore, because plants do not sequester their germline, these mutations can be transmitted to reproductive organs and subsequently to sexual offspring [14]. During clonal growth, somatic mutations that negatively impact sexual fitness are free to accumulate as long as they have little or no deleterious effect on clonal growth [5],[6]. This led us to hypothesize that long-lived clonal organisms might suffer senescence. Typically, senescence results from an age-related decline in the intensity of natural selection, which allows late-acting mutations to accumulate within a *population of individuals*. Long-lived clones, however, might senesce because somatic mutations that reduce sexual fitness accumulate within a *population of ramets* (i.e., within a clone). Importantly, we expect such clonal senescence to occur even when the intensity of natural selection does not decline with age, because selection on sexual fitness is absent during clonal growth. This is not true of traits like root growth, ramet production, average photosynthetic rates, hormone sensitivity, or even a clone's susceptibility to stress from the abiotic environment, all of which are likely to remain under selection within the clone. To test if senescence of sexual fitness occurs at the level of the clone, we asked whether older clones of *Populus tremuloides* (trembling aspen) exhibit lower reproductive performance. Specifically, we examined pollen production and viability among male clones from natural stands in British Columbia, Canada. Coupling molecular-based estimates of clone age with pollen data, we observed a significant decline in male fertility with increasing clone age.

*Populus tremuloides* is a dioecious tree that forms clones consisting entirely of male or of female ramets. Each ramet within a clone is capable of both sexual and asexual reproduction. Sexual reproductive maturity is reached between 10–20 y of age while asexual maturity is reached at 1 y [15]. Individual reproductive shoots produce inflorescences (catkins) that often have between 80 and 100 flowers [16]. Projections based on the size of intermountain aspen clones, formed from lateral root suckers, suggest that clones vary in size from 1.5 to 43.6 hectares and that some of the oldest clones might even be as old as one million years [17],[18]. Because size-based age estimates might be biased if local ecological conditions constrain growth, we instead estimated clone age by measuring the amount of neutral genetic diversity that had accumulated within each clone at 14 nuclear microsatellite loci (Materials and Methods) [19]. Because aspen is dioecious, we assayed the fertility of male clones by sampling whole catkins and quantifying pollen viability and pollen count (Materials and Methods). Thus, our measure of male fertility for each genet/clone was one component of male sexual fitness, the average number of viable pollen grains per ramet per catkin. Although we recognize that male sexual fitness includes other components such as pollen germination, tube growth, number of anthers, and number of flowers, as shorthand we use the terms male sexual fitness and male fertility interchangeably.

## Results

In a previous study, we measured the accumulation of neutral somatic mutations within 20 clones from Riske Creek, British Columbia, by genotyping 719 ramets at 14 microsatellite loci [19]. Because variation within a clone is expected to accumulate over time since initiation from a seed, we used genetic diversity within the *k* ^th^ clone (π*_k_*) to estimate the age of the clone [19].

**Figure 1:**
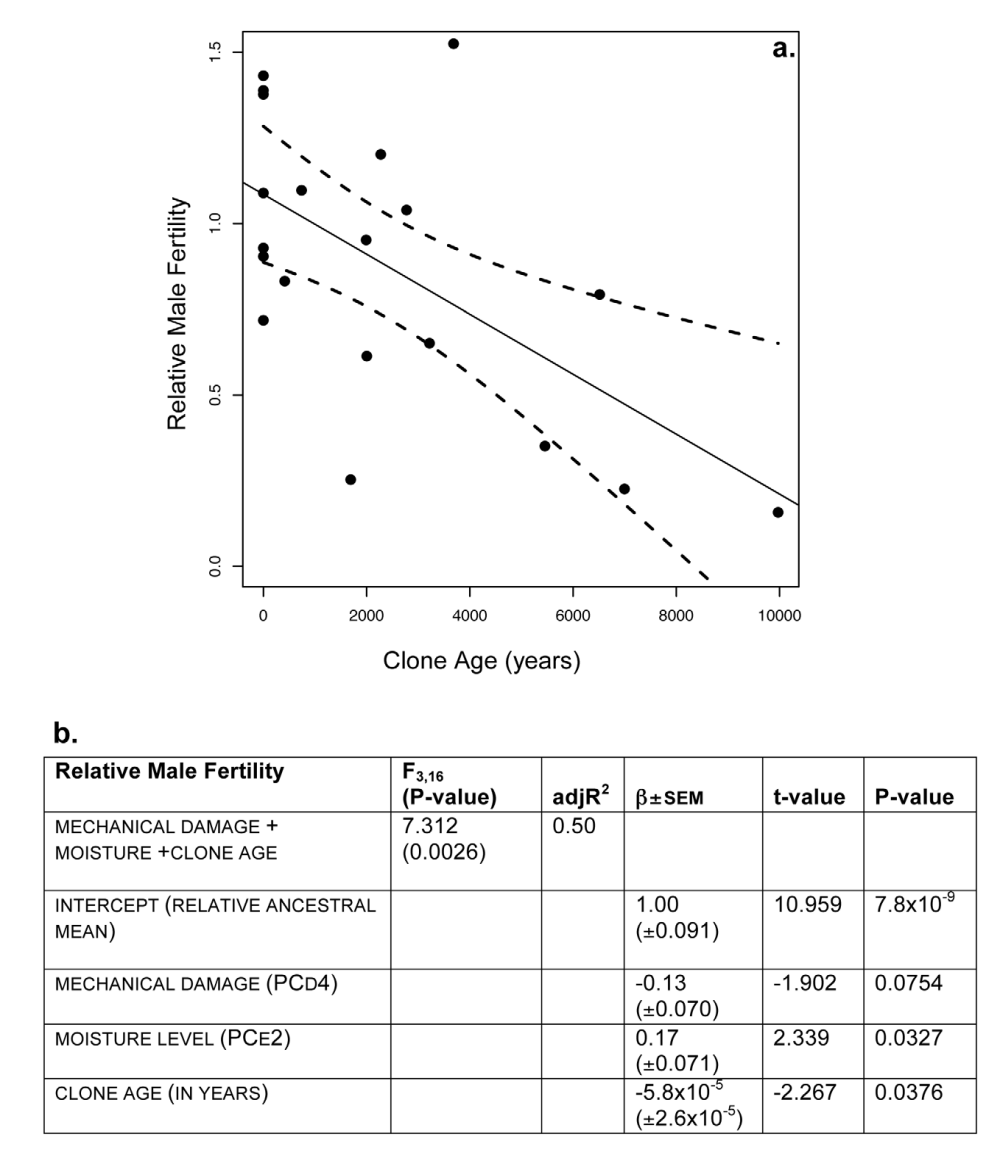
Male fertility declined significantly with increasing clone age. Relative clone sexual fitness was measured as the average number of pollen grains per catkin per ramet, divided by the estimated ancestral mean fitness (from the absolute value of the intercept of 17,456). In this figure we present both the results of a multiple regression and those from a simple linear regression with a single predictor, clone age, based on glacial calibration. (a) From a simple linear regression, we found the estimated slope was −8.1×10^−5^ (*F*_1,18_ = 10.41, *p* = 0.005, *R* ^2^ = 0.33, 95% CI: −1.3×10^−4^ to −2.8×10^−5^). Dashed curves represent the 95% confidence intervals around the fitted line. A randomization test confirmed the significance of this relationship (Figure S5, *p* = 0.025). (b) Results from a multiple linear regression confirm that male sexual fitness declines with clone age.

We found substantial variation among clones for male fertility, with older clones exhibiting significantly lower numbers of viable pollen than younger clones (Figure 1a,b). While our estimates of clone age are subject to error, we infer the same pattern in the raw data (Figure S6 and Figure S7): clones exhibiting more variation at microsatellite loci produce a significantly lower number of viable pollen grains per catkin per ramet. The observed variation in male sexual fitness among clones could not be explained by factors such as date of flower collection (*F*_2,94_ = 2.243, *p* = 0.11, *n* = 96) and inbreeding level (*F*_1,18_ = 1.142, *p* = 0.30, *R* ^2^ = 0.06, *n* = 19) (Text S1; Figures S1, S2, and S4). Furthermore, there was no relationship between ramet age and male sexual fitness, suggesting that ramet age plays a minor role relative to genet age with respect to senescence via the accumulation of mutations deleterious to male fertility (*F*_1,94_ = 3.801, *p* = 0.054, *R* ^2^ = 0.04, *n* = 95) (Figure S3). Empirical studies suggest that the presence of fungal pathogens and insect herbivory can exert a strong influence on reproductive success; thus we investigated the relationship between male fertility and these environmental factors. To quantify the effect of the variable of interest, clone age, on male sexual fitness, we performed a multiple linear regression accounting for the environmental factors that were substantially correlated with male sexual fitness. The best model consisted of three predictors: a composite measure reflecting the mechanical damage sustained by the average ramet in the clone (fourth principal component, PC_D_4), a composite measure reflecting the levels of moisture (second principal component, PC_E_2), and clone age (*F*_3,16_ = 7.312, *p* = 0.0026, adjusted *R* ^2^ = 0.50, Akaike Weight = 0.55) (Table 1, Figure 1c). Holding these environmental effects constant, average number of viable pollen grains per catkin per ramet again declined significantly with clone age (Figure 1, Figure S6, Text S1).

**Table 1:** Model comparison.

Model	Sexual Fitness	*F/p* Value	*df*		*AIC Criterion*	*ΔAIC*	*W*	*R^2^*
A	intercept only	—	1	19	411.76	11.311	<0.01	—
B	Clone Age	10.41/*p* = 0.005	1	18	404.63	4.15	0.07	0.33
C	PC_E_2 (moisture)	5.665/*p* = 0.028	1	18	408.28	7.80	0.01	0.40
D	PC_D_4 (mech damage)	4.05/*p* = 0.059	1	18	409.70	9.22	<0.01	0.14
E	moisture+clone age	7.94/*p* = 0.0040	2	17	402.57	2.09	0.19	0.42
F	mech damage+clone age	6.52/*p* = 0.010	2	17	404.37	3.89	0.08	0.37
G	mech damage+moisture	6.76/*p* = 0.00693	2	17	404.06	3.58	0.10	0.38
**H**	**mechdamage + moisture + cloneage**	**7.312/** ***p*** ** = 0.0026**	**3**	**16**	**400.48**	**0**	**0.55**	**0.50**

Three predictors showed a correlation greater than 0.3 in magnitude and were thus selected for inclusion in a multiple linear regression analysis: PC_E_2 (mechanical damage), PC_D_4 (moisture level), and clone age. In R, we used the function AIC(), which computes the Akaike Information Criterion using the formula *n*log(*RSS*/*n*)+2*p*, where *RSS* is the residual sum of squares and *p* is the number of parameters estimated. The *ΔAIC* is computed as the difference between the AIC values for each model and the model with the lowest AIC (Model I), excluding model C (based on different data). Model I is also selected to be the best fitting model using Akaike Weights (*W*), which measure the relative probability that the model is the best fit to the data tested [49].

**Figure 2:**
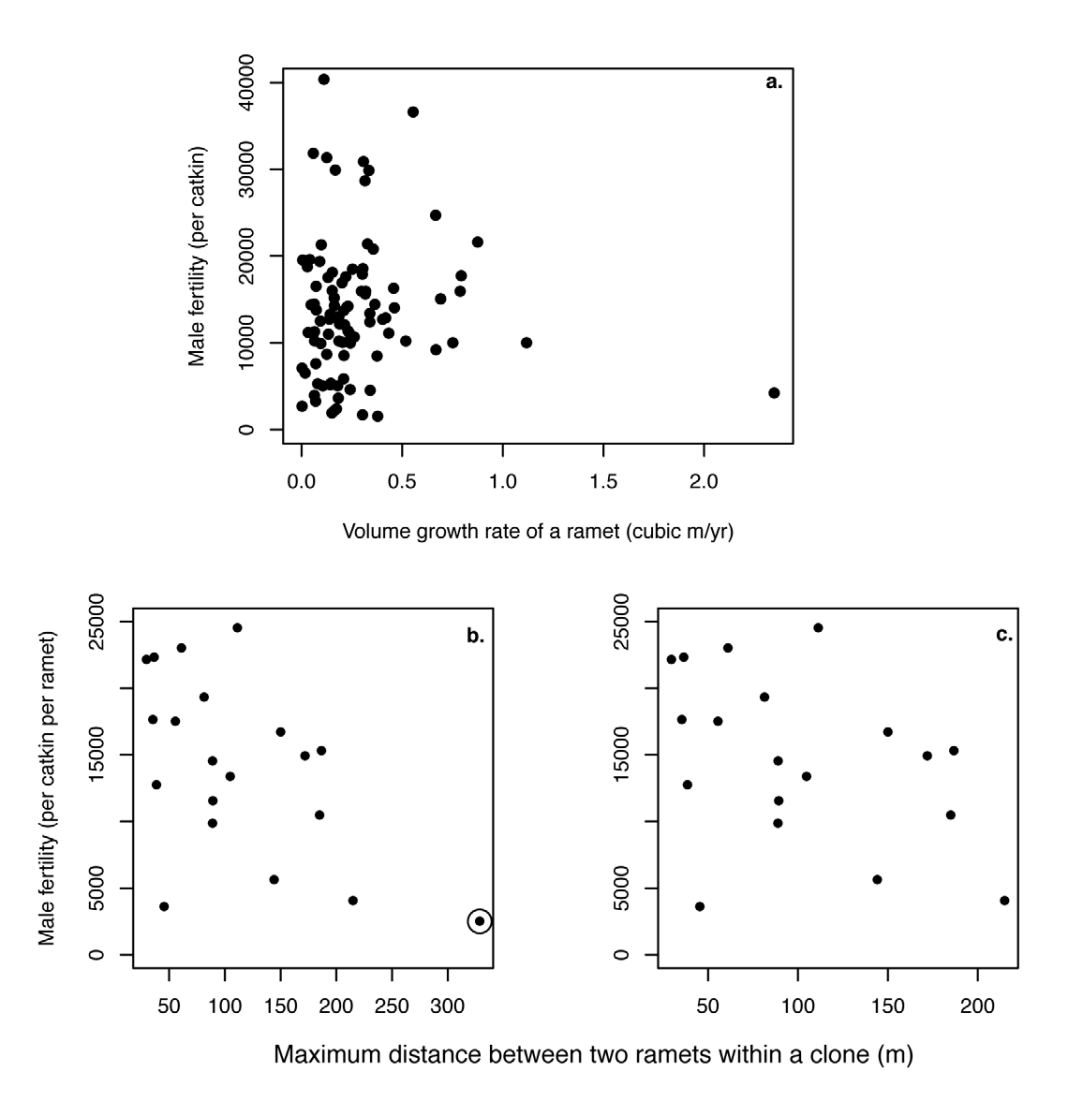
Male fertility and asexual fitness measures are not significantly related. (a) At the ramet level, there was no evidence for a trade-off between growth rate as measured by volume growth per year (*m* ^3^ per year) and ramet male fertility (*r* = 0.04, *t* = 0.388, *df* = 94, *p* = 0.70). (b) Although a correlation analysis between maximum distance between two ramets in a clone, D_max_, and the clone's mean male fertility was significant (*r* = −0.55, *t* = −2.762, *df* = 18, *p* = 0.013), this relationship was sensitive to the inclusion of a single data point (indicated by a circle). (c) Removing this point, the relationship is no longer significant (*r* = −0.40, *t* = −1.802, *df* = 17, *p* = 0.09).

If there were a trade-off between sexual and asexual function, selection could have facilitated the observed loss in male fertility with clone age because mutations deleterious to male sexual fitness would have increased vegetative success and risen in frequency during clonal growth. To investigate this possibility, we asked whether ramets with lower fertility exhibited higher asexual fitness, measured as the rate of increase in ramet volume per year. Specifically, we divided the volume of a ramet, *V* (in m^3^, obtained from the formula for a cylinder, V = π*h*(*d*/2)^2^, where *d* is the diameter at breast height and *h* is ramet height) by the age of the ramet from tree-ring data. We found no correlation between the male sexual fitness of male ramets and their growth rate (Figure 2a).

The accumulation of alleles that were beneficial to asexual growth might not have affected ramet growth but could have affected the rate of clonal expansion. Alternatively, the observed correlation between male sexual fitness and clone age could have been caused by genetic variation among clones (reflecting genetic variation among the seeds that established the clones), where some clones had higher asexual fitness and were more likely to survive for long periods of time but at a cost to male fertility. In either case, we would expect a negative relationship at the clone level between male fertility and asexual fitness. There was, however, no substantial correlation between male fertility and three potential measures of clone fitness: clone area (*r* = −0.33, *t* = −1.465, *df* = 18, *p* = 0.16), clone perimeter (*r* = −0.36, *t* = −1.662, *df* = 18, *p* = 0.11), and the maximum distance between any two ramets in the clone, D_max_ (Figure 2c). Furthermore, an analysis of variance indicated that much of the variation in clone fertility can be explained by clone age (or, equivalently, genetic diversity, *π_k_*) with very little attributed to the size of a clone (ANOVA: clone age: *F* = 11.55, *p* = 0.0034; clonal spread (D_max_): *F* = 2.972, *p* = 0.10). We also considered whether the accumulation of mutations reducing male fertility might be associated with an increased density of ramets within a clone. Our previous work [20] determined that a patch is largely comprised of a single clone with smaller clones near the edge, so we used estimates of the density of ramets within a 10 m×10 m square at the centre of each patch as a proxy for the density of ramets within a clone. There was, however, no significant relationship between density of ramets in a patch and male sexual fertility (*r* = −0.124, *t* = −0.5031, *df* = 16, *p* = 0.62). We caution that all of the above measures provide only rough estimates of clone fitness. To measure clone fitness accurately and to determine any trade-offs with sexual fitness would require a long-term common garden study examining the rates of clonal spread from seed. Thus, while we find no evidence that trade-offs (negative pleiotropy) explain the reduction in male fertility with clone age, we do not exclude this possibility.

## Discussion

Evidence that perennial plants exhibit demographic senescence is scarce because obtaining data on survivorship or fecundity from late-life perennials typically requires long-term demographic data. This proves difficult even in short-lived perennials. For example, to demonstrate aging in *Plantago lanceolata*, one study followed 30,000 individuals over 7 y, finding that, during times of stress, older-aged cohorts had significantly higher mortality rates relative to younger-aged cohorts [7]. Unlike many herbaceous perennials, most woody tree species are large in stature and have an extended life cycle, rendering such experiments impractical. Although our data are not without their caveats and limitations, our work offers a novel approach for obtaining late-life demographic data on a variety of clonal species by using a molecular clock to age individual clones.

We observed a significant decline in male fertility with clone age (Figure 1), causing a reduction of 8% in the average number of viable pollen grains per catkin per ramet, on average, among the clones sampled. Given the maximum age of the oldest clone was ∼10,000 y based on glacial history in this region of British Columbia, we estimate that the rate of decline in average number of viable pollen grains was 5.8×10^−5^ per year (95% CI: 3.8×10^−6^ to 1.1×10^−4^ based on the multiple linear regression, Figure 1c). Given a minimum age of the youngest clones of 71 y based on tree ring data, the estimated decline was 1.6×10^−3^ per year (95% CI: 1.03×10^−4^ to 3.1×10^−3^). Assuming a constant linear decline, it would thus take between ∼500 and 20,000 y for a clone to lose sexual function with respect to pollen quantity and quality.

A plausible explanation for the observed decline in male sexual fitness with increasing clone age is that somatic mutations that negatively impact pollen fitness accumulate over time. As is the case with meiotic mutations, somatic changes that arise during mitosis can be neutral, deleterious, or beneficial. While somatic selection among cell lineages would act to eliminate deleterious somatic mutations, those mutations that have little to no effect on clonal growth but that reduce sexual fitness are free to accumulate. As mutations affecting fitness tend to be deleterious and partially recessive, at least some somatic mutations may be largely masked in the diploid clone phase but be deleterious in the haploid pollen stage, reducing pollen fitness among older clones. Although we observed a higher number of somatic mutations at microsatellite marker loci among the clones that exhibit reduced male fertility (Figure 1, Figure S6, Figure S7), we have no reason to expect these marker loci are directly responsible for the observed declines in sexual fitness. These marker loci only confirm that somatic mutations can and do accumulate. Two previous studies on clonal ferns showed a direct link between somatic mutations and reduced fitness, using segregation patterns of deleterious mutations among gametophytes obtained from fern clones [21],[22]. In long-lived plant taxa where higher per generation mutation rates are often found ([6],[23],[24] but see [25]), post-zygotic somatic mutations may contribute substantially to the total mutation rate and genetic load [25],[26].

An alternative explanation is that somatic mutations reducing sexual function have spread within these clones because they enhance clonal fitness (negative pleiotropy), for example, due to trade-offs in resource allocation. We looked for evidence of such trade-offs at two levels: ramet and clone. We found no evidence of a relationship between male fertility and volumetric growth per year (m^3^/y) of a ramet (Figure 2a). Additionally, a trade-off at the level of the clone might predict that larger-sized clones (regardless of clone age) should exhibit a reduced sexual fitness when compared to smaller-sized clones, due either to the accumulation of somatic mutations that enhance clonal spread and/or to genetic variation among the seeds that established the clones. We did not, however, find any significant correlations between sexual fitness and three potential measures of clone size/fitness (Figure 2c). Nor was there any evidence that clone size was related to clone age [19]. Although we did not detect evidence for negative pleiotropy, we cannot rule out the possibility that the loss of sex in aspen was driven by the spread of beneficial mutations that improve cell- or ramet-proliferation.

A final alternative explanation for why older clones exhibit lowered reproductive performance is that heritable epigenetic changes accumulate that reduce sexual traits. It has been shown previously that allopolyploidization, a change in reproductive mode, and nutritional stresses can lead to both genetic and epigenetic re-patterning [27]. Furthermore, there is growing evidence that epigenetic mechanisms like DNA methylation and siRNAs are responsible for natural population variation in traits like flower symmetry, self fertility, flower initiation, and number of reproductive organs [27]. Although epigenetic mechanisms like paramutation may be highly stable [28], it is unknown if such heritable epigenetic changes could persist over hundreds to thousands of years and over multiple ramet generations.

With current advances in sequencing technologies, it will become increasingly cost-effective to assess the age of clones using a molecular clock and to ask whether sexual fitness declines with clone age as we have found in trembling aspen. Furthermore, given that previous work has shown an increased transmission of deleterious mutations through the sperm than egg [29], it would be interesting to assess whether female versus male clones differ in the amount of senescence that they exhibit.

In long-lived perennials and clonal plants, substantial numbers of somatic mutations can accumulate over time [6],[13],[23],[24],[30]. This is because in plants there is no distinction between the soma and germline. Somatic cell lineages are not protected in a quiescent non-replicative state and can actively divide, eventually giving rise to gametes whenever reproductive tissues form. Although somatic mutations need not be immediately life-threatening, they can have a devastating impact on sexual function when they are unmasked in the haploid state. This suggests that, in the face of accumulating somatic mutations, plant clones may have a limited time span within which sexual function remains high. The aspen clones that we examined have lost, on average, 8% of their fertility, with less than a quarter of the pollen fertility remaining in the oldest clone (Figure 1). Without sex, clones of *Populus tremuloides* are unable to disperse beyond their immediate local environment. Our data provide evidence that male fertility declines with advancing age, demonstrating that aging is inevitable in aspen clones.

## Materials and Methods

### Study Sites and Sampling Strategy

We collected foliage for microsatellite analysis from 871 trees of *Populus tremuloides* sampled from two populations in Canada: Riske Creek, British Columbia (*N*_clones_ = 20, *N*_ramets_ = 719), and Red Rock, Waterton Lakes National Park, Alberta (*N*_clones_ = 29, *N*_ramets_ = 152). Trees on the perimeter and along transects were physically mapped using both a measuring tape and a handheld Global Positioning System (GPS) unit. Details on the Red Rock population are not included here because this mountainous population was comprised of very small clones. The foliage from trees/ramets were sampled in two ways: on the perimeter of a patch and systematically along two or three 30–50 m transects within the patch. On average 30–50 individuals were sampled per patch. No tree less than 1.5 m in height was sampled, and only patches separated by at least 1 km of terrain lacking aspen trees were used. We physically mapped ramets, measured height and diameter at breast height on all ramets, and took an increment core from a sample of ramets belonging to each genotype.

### Clone Age Estimation

Estimates of clone age in years are detailed in Ally et al. (2008) [19]. In short, if neutral mutations accumulate in a clock-like manner at such loci as microsatellites, then coupling the amount of molecular diversity within a clone (*π_k_*) with a mutation rate (*μ*) can provide a measure of a clone's age. We examined 14 microsatellite loci for somatic mutations across 719 ramets in 20 clones. We scored an allele as a somatic mutation if an individual ramet in a clone differed by one allele at one locus but shared the same alleles at all other loci as the most frequent genotype. Somatic mutations were counted only if we were able to confirm their presence with two subsequent PCRs on the same ramets. Because we found that mutations accumulated within a clone in a manner consistent with a star-like phylogeny [19], the probability that a mutation had accumulated at a locus in either of two ramet lineages is expected to equal 4 *μT_CCA_*. Here, *T_CCA_* represents the clone age or the time to the common cellular ancestor, the seed, and 2 *μ* is the mutation rate per diploid ramet per locus per year [31]. Clone age can thus be estimated from the pairwise genetic distance, *π_k_*, averaged over all pairs of ramets and all loci within the *k^th^* clone. This assumes that the ramets accumulate somatic mutations according to a star-like phylogeny, i.e., independently. In our study, sampled ramets were well spaced from one another, with an average distance between any two ramets of 38 m (s.e. = 3.31 m). Although somatic mutations were occasionally shared by neighboring ramets, this affected only a small number of pairwise comparisons within a clone. Furthermore, we have shown theoretically that the relationship between *T_CCA_* and *π_k_* is robust to small departures from a star-like coalescent history, allowing for the possibility that some ramets are more closely related [19].

We thus estimated the time since initiation of the clone, *T_CCA_*, as *π_k_*/(4 *μ*). Rather than estimating the mutation rate directly, we obtained upper and lower bounds based on the minimum and maximum possible ages of the clones. To estimate the youngest age any clone could be, we used tree ring data, reasoning that a clone had to be at least as old as the oldest cored ramet. This provided an upper bound on the mutation rate per year, *μ_upper_*, by setting the average value of *π_k_* across clones to 4*μ* times the average age of the oldest cored ramets (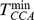 = 71 y), yielding 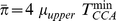. Here, we used all clones except the most divergent clone (which was likely much older than the oldest cored ramet). Given the neutral genetic diversity within the *k^th^* clone (*π_k_*), this upper bound on the mutation rate was used to estimate the absolute minimum age of each clone, 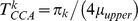. Similarly, the oldest that a clone could be is 10,000 y old. According to the glacial history of British Columbia, this is when the ice sheets retreated from the study area [32]. We thus obtained a lower bound estimate for the mutation rate, *μ_lower_*, by setting the age of the clone with the most diversity (*π*_max_ = 0.0335) to 10,000 y and using 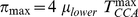 to solve for *μ_lower_*. Upper and lower bound estimates of the microsatellite mutation rate were thus *μ_upper_* = 2.3×10^−5^ and *μ_lower_* = 8.4×10^−7^ [19].

### Estimating Male Fertility

In *Populus tremuloides*, clones are either male or female; we chose to focus only on male clones, whose fitness components were more readily measured. In contrast, many plant evolutionary studies use monoecious plants with male and female organs on the same individual [7],[33]–[36]. In such cases, it is possible to measure all aspects of sexual function each generation, including pollen and ovule fitness. Aspen catkins produce between 50 and 100 flowers per inflorescence, with each male flower containing approximately 7–11 anthers [37]. Recognizing the limits of field-based measures, we thus treat the average number of viable pollen grains per catkin per ramet as a proxy for the potential of each male clone to produce further sexual offspring.

In the spring of 2003, we collected 5 whole catkins from each individual ramet, sampling 4–6 ramets per clone in Riske Creek (*N*_ramet_ = 97, *N*_clone_ = 20). Every attempt was made to ensure that the catkins had flowers that were fully open and that functional anthers were in the two-lobed condition, indicative of a staminate flower just prior to the shedding of pollen [38],[39]. To determine if, at the time of collection, the degree of flower/catkin development affected our estimates of male fertility, we noted the state of the catkin (see Figure S2 for state descriptions). Attempts were made to collect replicate catkins from different parts of the ramet crown. Given time constraints and the small size of individual flowers, we did not separate out anthers and suspend them in a mixture of lactophenol-aniline blue as is typical of pollen viability studies. Instead, whole catkins were put immediately into a tube containing lactophenol-aniline blue [40], and a pestle was used to mechanically free the pollen grains from the anthers. If during the mechanical mixing of anthers (from a single catkin) not all anthers were physically opened, then we are likely to have detected fewer pollen grains per catkin. This is, however, a systematic sampling error that should be present across all samples. All sample tubes were assigned a randomly generated code. These were then brought back to the lab where pollen counts and estimates of the proportion of viable pollen were assayed by two “blind observers.” A pollen grain that was unstained, collapsed, and abnormally shaped was considered non-viable. Pollen count for each ramet was estimated from a sample using a Neubauer hemocytometer and a microscope (4× objective). Estimates of the proportion of viable pollen grains were made on a standard microscope slide (at 40×), making three sweeps lengthwise along the slide and counting both viable and non-viable pollen. The average number of pollen grains counted on a slide was 1,756.

Thus, mean male fertility was a composite measure that included pollen viability and pollen count. We acknowledge that our composite measure only captures some components of male sexual fitness. In controlled breeding trials where dormant floral branches are collected and then forced to flower in greenhouses, it may be relatively easy to measure additional fitness components like pollen tube growth and pollen germination. This was not possible in our field-based study.

### Assessing the Abiotic and Biotic Environment of Clones

Empirical studies suggest that plant sexual and asexual reproductive success is affected by the presence of fungal pathogens and insect herbivory [41]–[45]. Thus, for all sampled trees, we measured 11 morphological variables that have been shown previously to reflect disease status for *P. tremuloides* trees [46],[47]: diameter at breast height (cm), height (m), number of conks, number of cavities, percentage of dead branches in crown, presence/absence of sap, number of scars, average length of scar (cm), proportion of leaves scored as eaten, proportion of leaves with a gall, and proportion of leaves exhibiting a leaf minor.

A second aspect of the environment affecting plant reproductive success is site quality, which reflects available resources like soil moisture, nutrients, drainage level, light, and soil temperature. Environmental variables like moisture vary through time, and thus accurate and detailed assessments of site quality can be time consuming, expensive, and difficult to obtain. As a proxy for site quality, plant assemblages are often used because indicator plant species reflect differential resource availability and the form of competitive interactions. Previous work on aspen-dominated communities has indeed shown that understory vegetation was significantly correlated to site quality [48]. Thus, to obtain an assay of site quality, we measured the percent cover of trees, shrubs, herbaceous plants, moss, lichens, and bryophytes in the centre of each patch using a sampling plot with a radius of 5 m. Surveys were conducted during the months of May and July 2003. Where possible, all non-woody herbs and shrubs were identified to species level. In a few cases where habitats were similar and more than one species of a genus was found, we collapsed species into genus-level groups to reduce the number of variables in our analyses. In addition, we dug soil pits in each of the sampled patches, and from a combination of topographic and soil morphological properties, we obtained data on soil moisture regime, soil nutrient regime, and drainage class [48]. These soil characteristics were recoded into binary data for the patch.

### Model Selection

We performed two separate principal component analyses (PCA) because our variables reflect different physical scales as well as different aspects of ecology. Specifically, our morphological variables were measured for every sampled tree and indicate susceptibility to disease and health of the individual ramet, while site variables reflect the environment found within a clone. Eleven different ramet health variables were reduced to four composite measures, while plant understory cover and abiotic site variables were reduced to eight environmental indices using principal component analysis (Supporting Information).

To assess the relevance of these abiotic and biotic variables to mean sexual fitness, we first examined the data using Pearson product moment correlations (*r*) and scatterplots. No correction was made for multiple comparisons because we were simply identifying potential predictors. From this we chose only those predictors that showed a sizable correlation with sexual fitness (*r*>|0.30|). The predictors that showed a correlation greater than |0.30| were PC_D_4: mechanical damage (*r* = −0.43), PC_E_2: moisture (*r* = 0.43), and the predictor of interest, clone age (*r* = −0.56). We calculated the Akaike Information Criterion (*AIC*) and then obtained the Akaike Weight (*W*) to determine the relative probability that a given model best fit the data (Table 1) [49]. These variables were then put into a stepwise multiple regression analysis. Subsequent model selection was based on AIC criterion, *p* values, and adjusted *R* ^2^ values.

## Supporting Information

Figure S1 **A histogram showing the variation in mean male fertility in the Riske Creek population.** Mean male fertility in Riske Creek was 13,647 viable pollen grains per catkin (s.d. = 7,834; *N*_ramet_ = 97).
(0.44 MB TIF)

Figure S2 **State of catkin emergence at collection time did not explain male fertility variation in Riske Creek.** The following factors were used in an ANOVA: partially emergent catkins, mostly emergent, or catkins completely open but anthers have not yet dehisced. Here, we show the raw sexual fitness data plotted against state of catkin emergence. Horizontal lines are the means for each factor. Given the skew in the data, we performed an ANOVA on the square-root transformed sexual fitness (*F*_2,94_ = 2.243, *p* = 0.11). An ANOVA on the untransformed data, however, yielded similar conclusions: *F*_2,94_ = 2.529, *p* = 0.085.
(0.36 MB TIF)

Figure S3 **Ramet age does not explain significant variation in average number of viable pollen grains.** Male fertility was measured as the average number of pollen grains per catkin. (a) The results from a linear regression against ramet age (*N* = 95) were: *R* ^2^ = 0.04, *F*_1,94_ = 3.801, *p* = 0.054. (b) Removing the oldest ramet as a potential outlier does not alter this conclusion: *R* ^2^ = 0.00013, *F*_1,93_ = 0.0127, *p* = 0.91.
(0.37 MB TIF)

Figure S4 **Inbreeding depression does not explain significant variation in average number of viable pollen grains.** (a) A Pearson's correlation test showed that the proportion of homozygous loci in a clone does not change with clone age, as measured by genetic diversity, (H_a_: *r*>0, *r* = −0.037, *t* = −0.1566, *df* = 18, *p* = 0.56). (b) With the oldest clone removed as a potential outlier, inbreeding level still does not change with clone age (H_a_: *r*>0, *r* = −0.049, *t* = −0.2028, *df* = 17, *p* = 0.58). (c) The level of inbreeding, as measured by proportion of homozygous loci, does not explain variation in the average number of pollen grains per ramet (*R* ^2^ = 0.06, *F*_1,18_ = 1.142, *p* = 0.30).
(0.38 MB TIF)

Figure S5 **Results from a randomization test on the association between mean clone male fertility and clone age.** Male fertility was sampled with replacement and randomly allocated to a given clone age. Spearman's rank correlation, *rho*, was used because it is the most conservative test of the relationship between age and sex because no assumptions are made about the frequency distributions of the variables involved. The total number of randomizations performed was 10,000.
(0.28 MB TIF)

Figure S6 **Relative male fertility declined as a function of increasing molecular diversity,** ***π_k_*** **, within a clone.** Relative male fertility was measured as the average number of pollen grains per catkin per ramet divided by the estimated ancestral mean fitness (the absolute value of the intercept = 17,456±1,599). A linear regression with a single predictor, *π_k_*, found the slope was −23.98±7.43 (95% CI: −39.60 to −8.37; *F*_1,18_ = 10.41, *p* = 0.005, *R* ^2^ = 0.33).
(0.29 MB TIF)

Figure S7 **Average number of viable pollen grains per catkin per ramet as a function of the number of observed neutral somatic mutations across 14 microsatellite loci.** (a) Male fertility declines with the number of somatic mutations observed within a clone (*F*_1,18_ = 15.04, *p* = 0.0011, β = −0.230±0.06). Assuming that older clones have had more time to accumulate somatic mutations, this figure shows that male fertility declines with clone age, without having to estimate age explicitly. Error bars represent the standard errors around the mean estimate. (b) Neutral somatic mutations were randomly allocated to different clones and a linear regression was performed on the randomized datasets. The observed slope of β = −0.230 (indicated by an arrow) was recovered in only 0.18% of the 10,000 randomized datasets.
(0.61 MB TIF)

Text S1 **We provide additional details on methods and data analysis.**
(0.06 MB DOC)
