# Supplementary figures and images for: Aging in a Long-Lived Clonal Tree
Source: PLoS Biol. 2010 Aug 17;8(8):e1000454. doi: 10.1371/journal.pbio.1000454 (PMC2923084; doi:10.1371/journal.pbio.1000454)

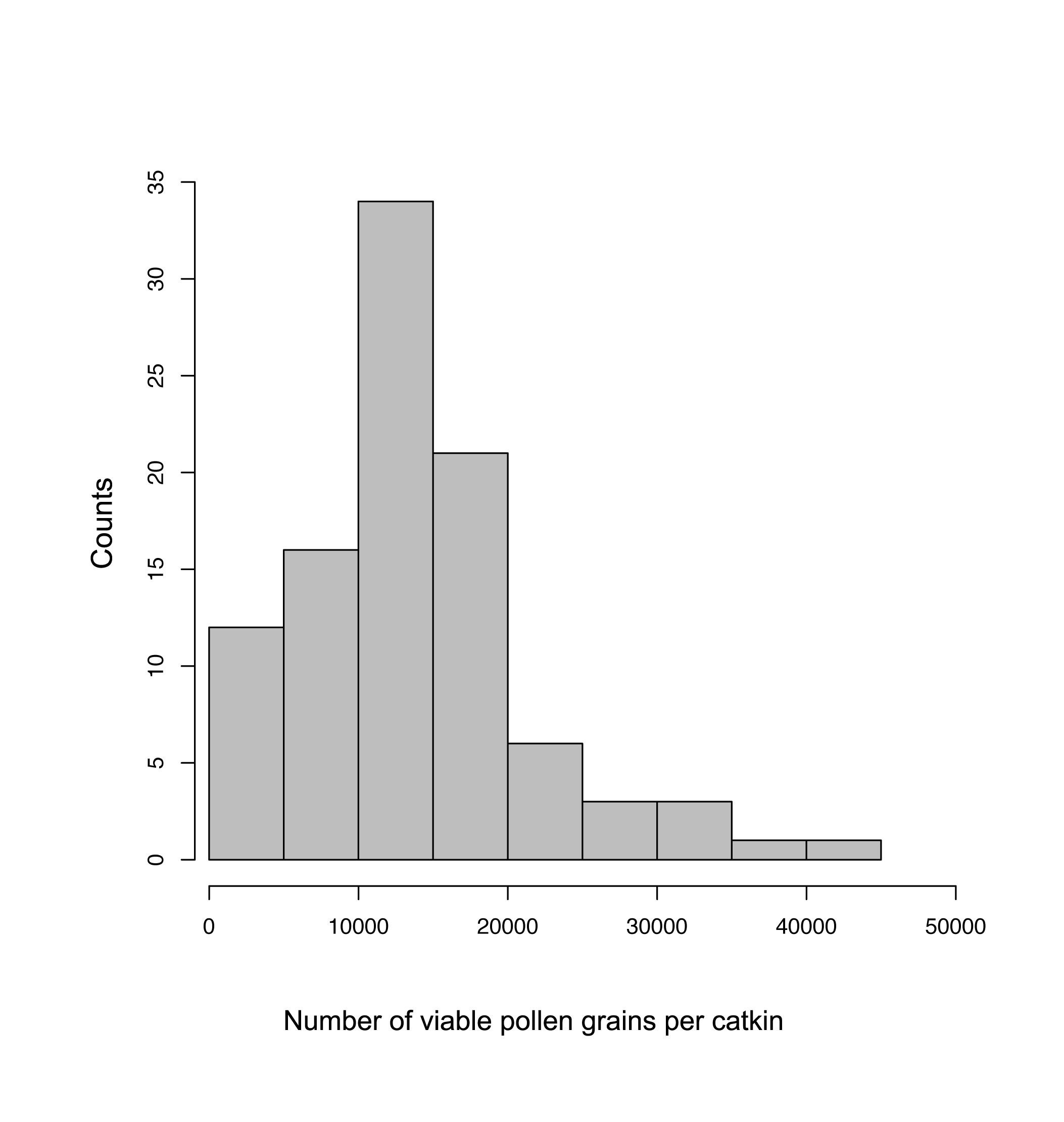

Supplement: Figure S1 — A histogram showing the variation in mean male fertility in the Riske Creek population. Mean male fertility in Riske Creek was 13,647 viable pollen grains per catkin (s.d. = 7,834; N ramet = 97). (0.44 MB TIF) [file pbio.1000454.s001.tif]

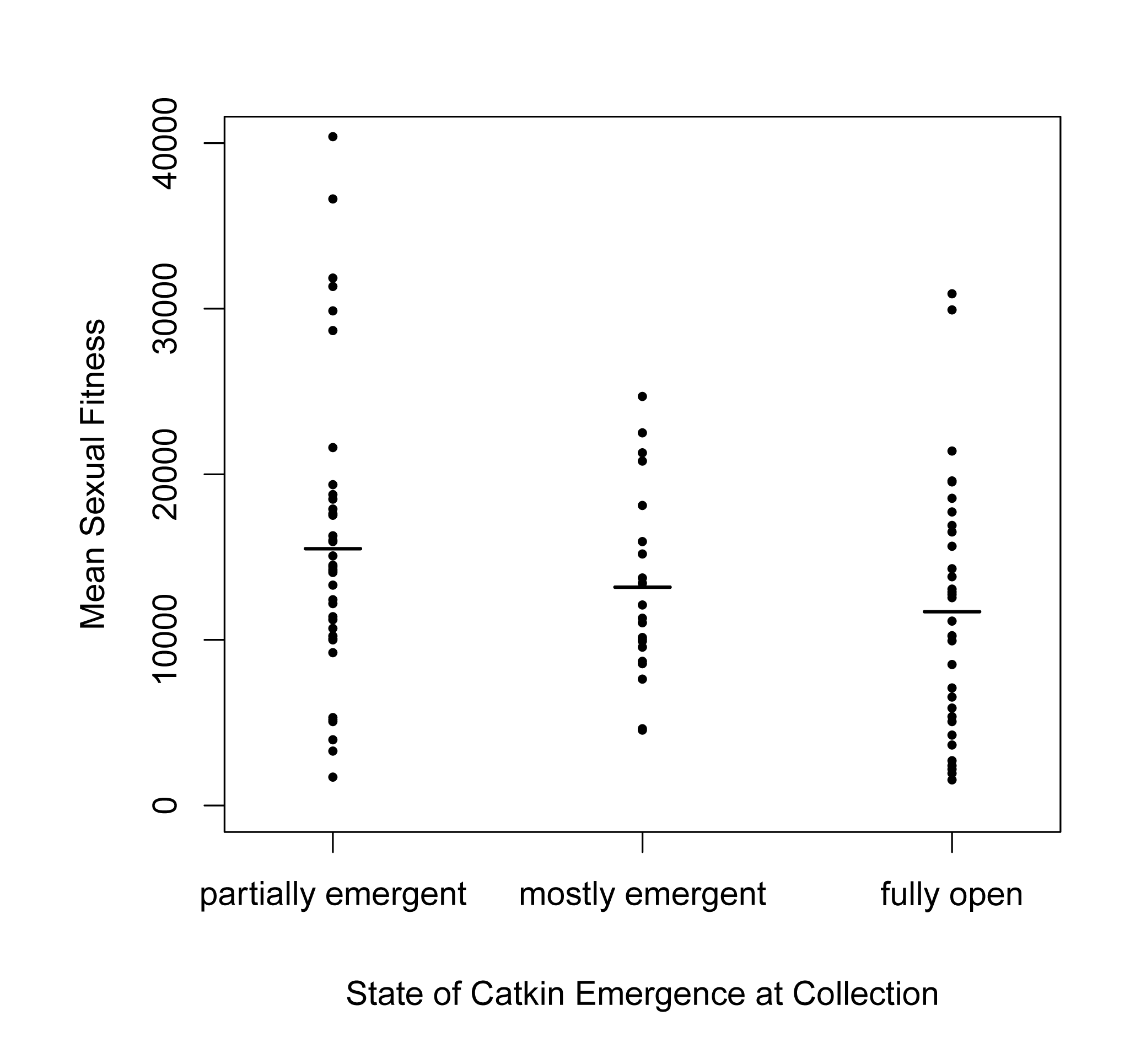

Supplement: Figure S2 — State of catkin emergence at collection time did not explain male fertility variation in Riske Creek. The following factors were used in an ANOVA: partially emergent catkins, mostly emergent, or catkins completely open but anthers have not yet dehisced. Here, we show the raw sexual fitness data plotted against state of catkin emergence. Horizontal lines are the means for each factor. Given the skew in the data, we performed an ANOVA on the square-root transformed sexual fitness (F 2,94 = 2.243, p = 0.11). An ANOVA on the untransformed data, however, yielded similar conclusions: F 2,94 = 2.529, p = 0.085. (0.36 MB TIF) [file pbio.1000454.s002.tif]

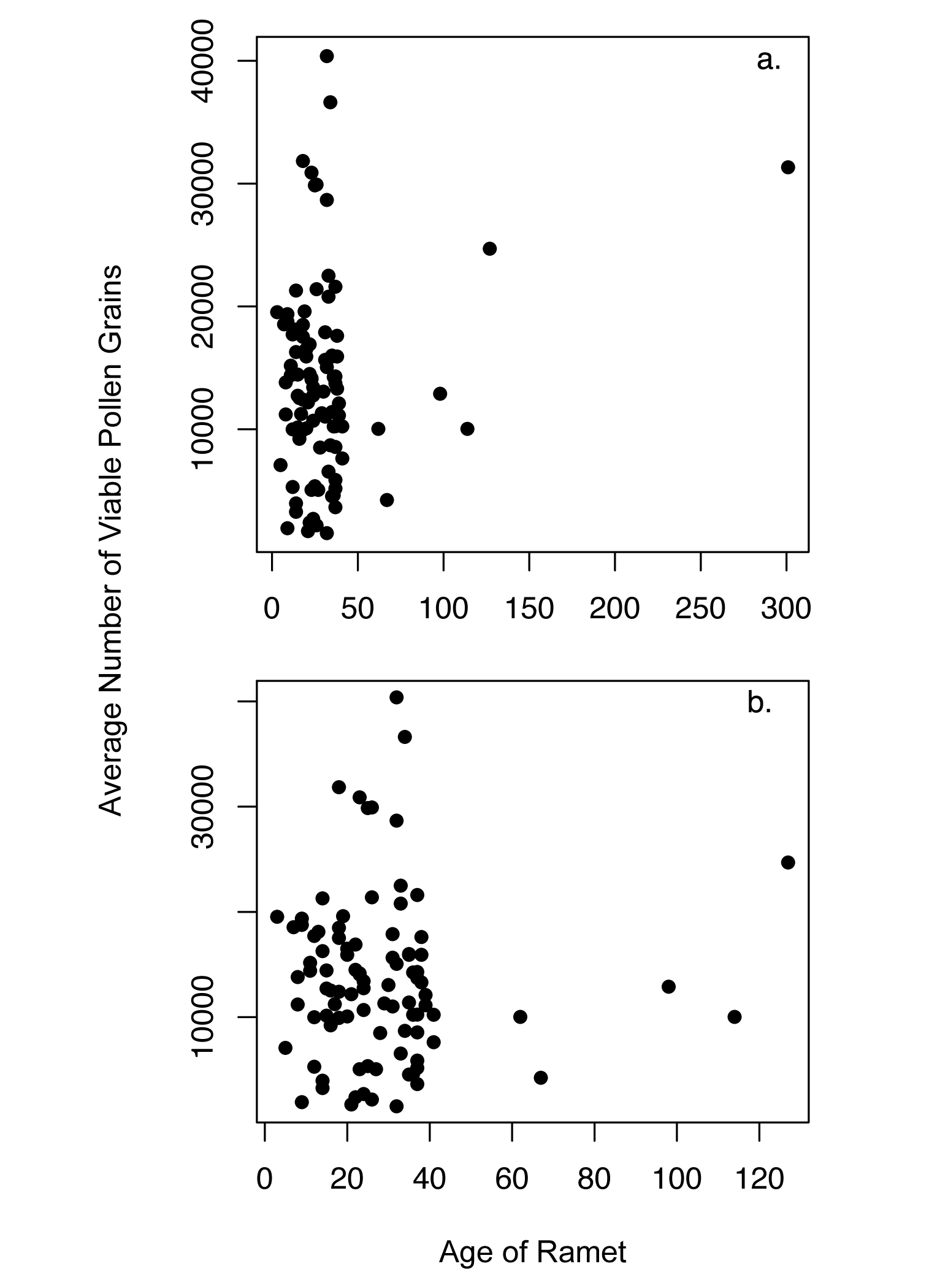

Supplement: Figure S3 — Ramet age does not explain significant variation in average number of viable pollen grains. Male fertility was measured as the average number of pollen grains per catkin. (a) The results from a linear regression against ramet age (N = 95) were: R 2 = 0.04, F 1,94 = 3.801, p = 0.054. (b) Removing the oldest ramet as a potential outlier does not alter this conclusion: R 2 = 0.00013, F 1,93 = 0.0127, p = 0.91. (0.37 MB TIF) [file pbio.1000454.s003.tif]

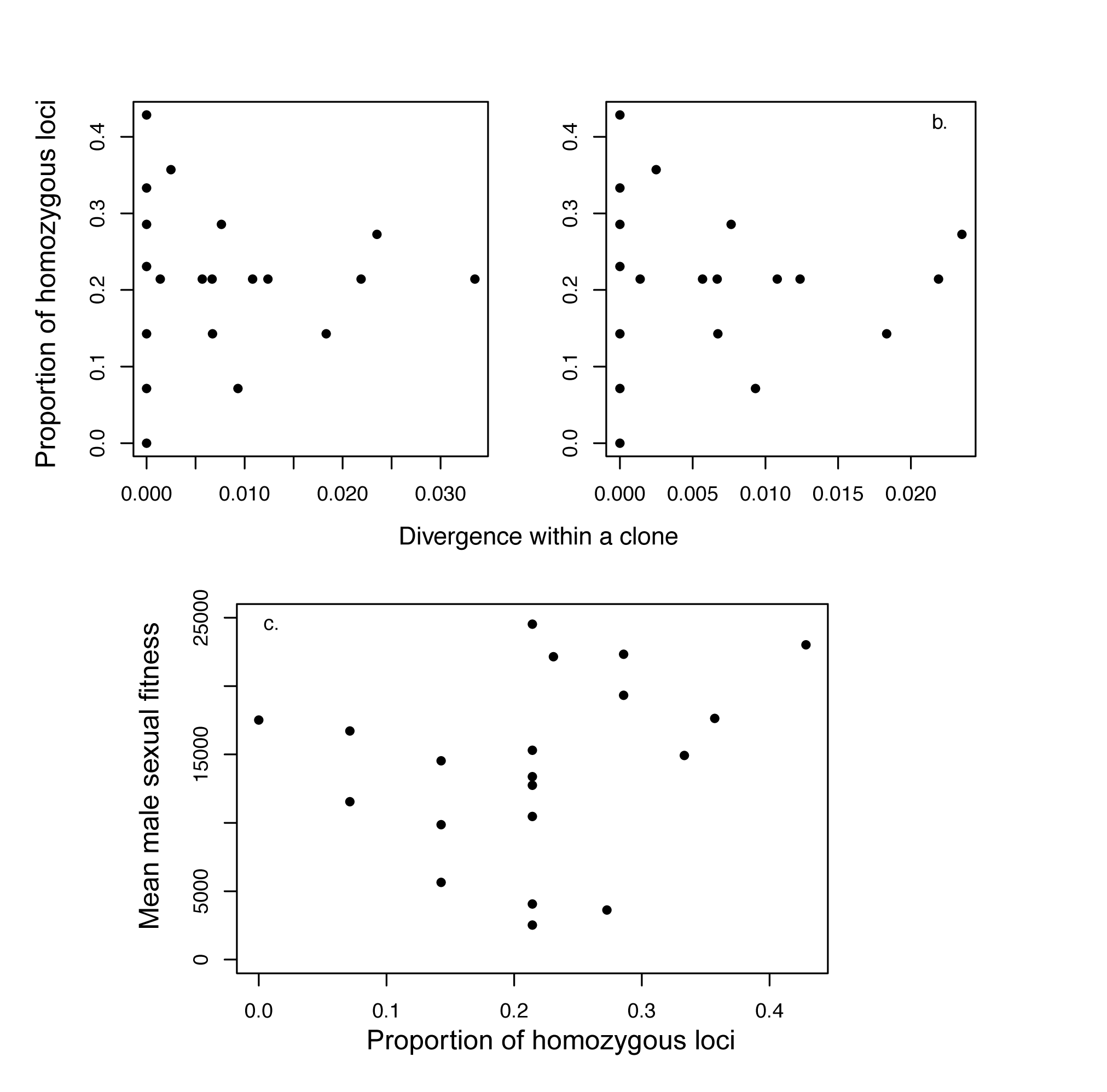

Supplement: Figure S4 — Inbreeding depression does not explain significant variation in average number of viable pollen grains. (a) A Pearson's correlation test showed that the proportion of homozygous loci in a clone does not change with clone age, as measured by genetic diversity, (Ha: r>0, r = −0.037, t = −0.1566, df = 18, p = 0.56). (b) With the oldest clone removed as a potential outlier, inbreeding level still does not change with clone age (Ha: r>0, r = −0.049, t = −0.2028, df = 17, p = 0.58). (c) The level of inbreeding, as measured by proportion of homozygous loci, does not explain variation in the average number of pollen grains per ramet (R 2 = 0.06, F 1,18 = 1.142, p = 0.30). (0.38 MB TIF) [file pbio.1000454.s004.tif]

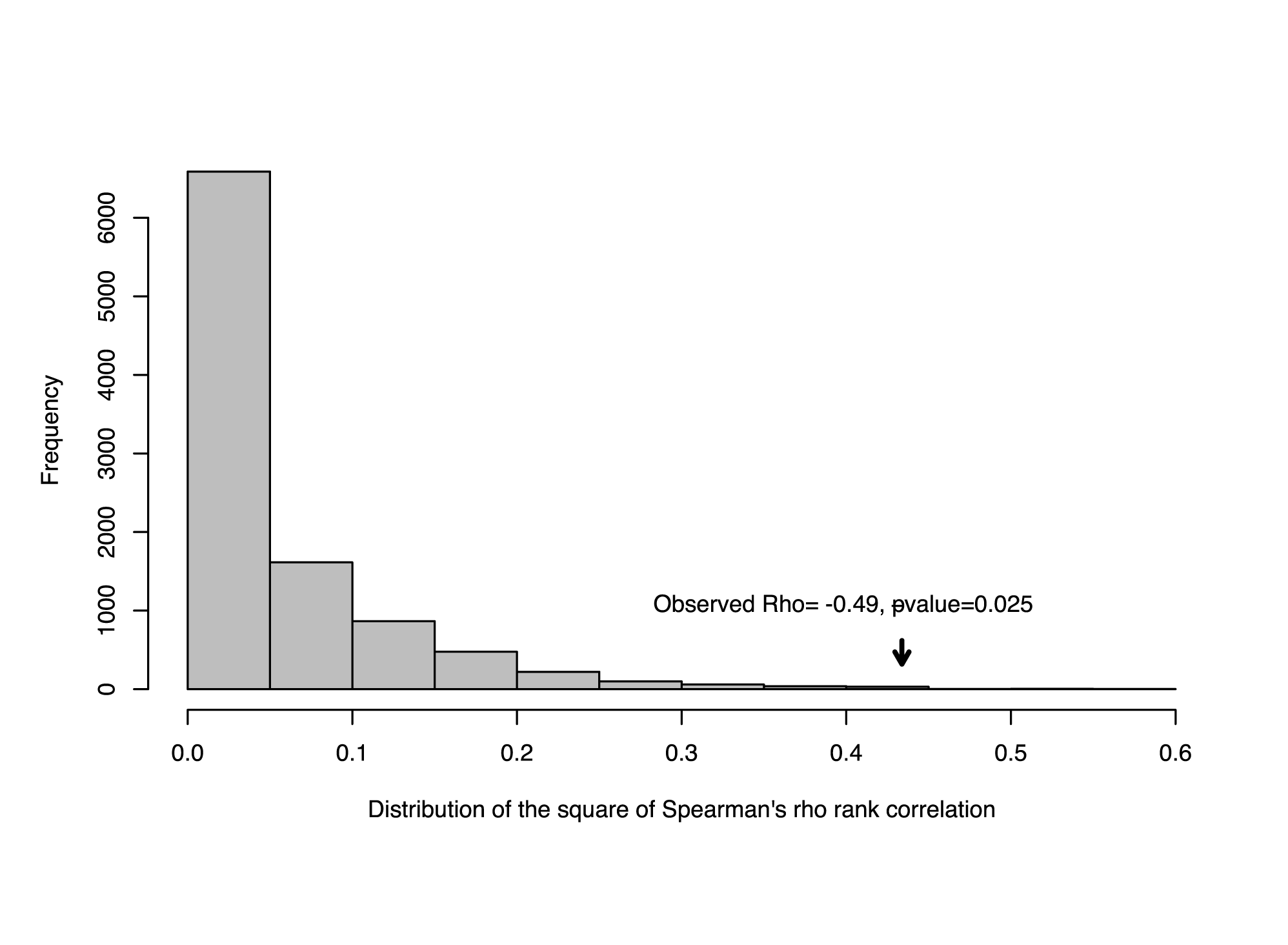

Supplement: Figure S5 — Results from a randomization test on the association between mean clone male fertility and clone age. Male fertility was sampled with replacement and randomly allocated to a given clone age. Spearman's rank correlation, rho, was used because it is the most conservative test of the relationship between age and sex because no assumptions are made about the frequency distributions of the variables involved. The total number of randomizations performed was 10,000. (0.28 MB TIF) [file pbio.1000454.s005.tif]

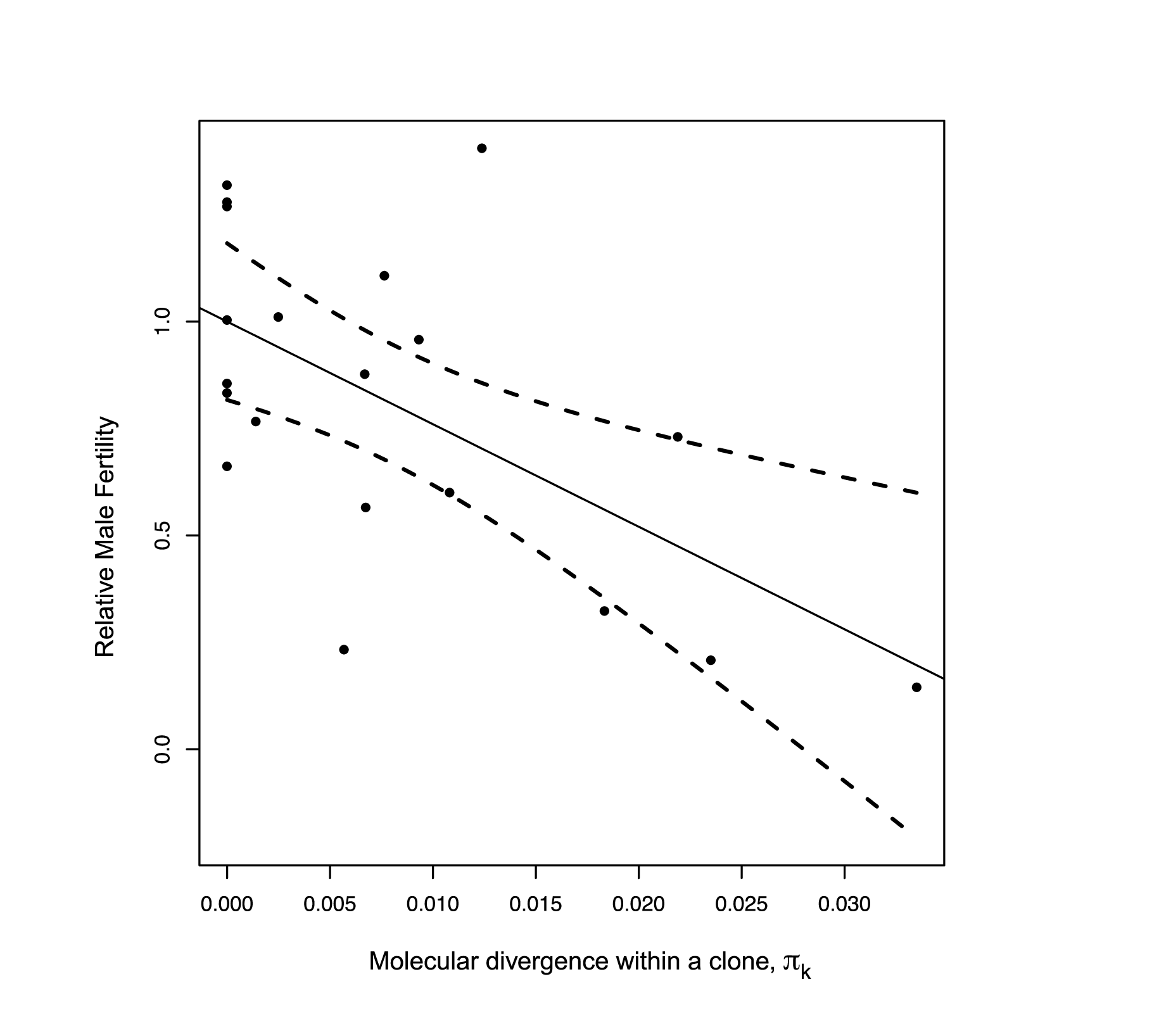

Supplement: Figure S6 — Relative male fertility declined as a function of increasing molecular diversity, πk , within a clone. Relative male fertility was measured as the average number of pollen grains per catkin per ramet divided by the estimated ancestral mean fitness (the absolute value of the intercept = 17,456±1,599). A linear regression with a single predictor, πk, found the slope was −23.98±7.43 (95% CI: −39.60 to −8.37; F 1,18 = 10.41, p = 0.005, R 2 = 0.33). (0.29 MB TIF) [file pbio.1000454.s006.tif]

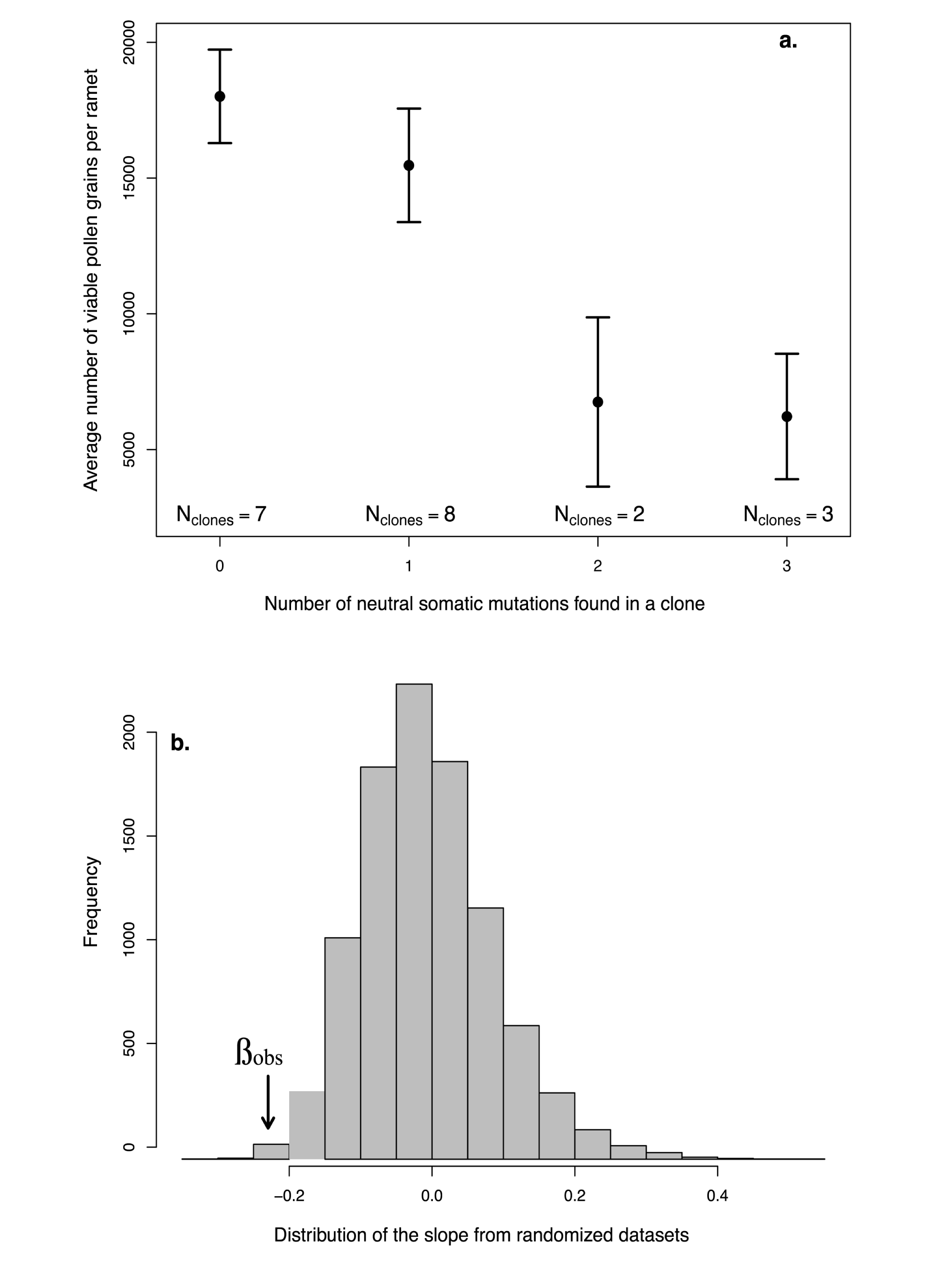

Supplement: Figure S7 — Average number of viable pollen grains per catkin per ramet as a function of the number of observed neutral somatic mutations across 14 microsatellite loci. (a) Male fertility declines with the number of somatic mutations observed within a clone (F 1,18 = 15.04, p = 0.0011, β = −0.230±0.06). Assuming that older clones have had more time to accumulate somatic mutations, this figure shows that male fertility declines with clone age, without having to estimate age explicitly. Error bars represent the standard errors around the mean estimate. (b) Neutral somatic mutations were randomly allocated to different clones and a linear regression was performed on the randomized datasets. The observed slope of β = −0.230 (indicated by an arrow) was recovered in only 0.18% of the 10,000 randomized datasets. (0.61 MB TIF) [file pbio.1000454.s007.tif]
