# Supplementary material for: Aging in a Long-Lived Clonal Tree
Source: PLoS Biol. 2010 Aug 17;8(8):e1000454. doi: 10.1371/journal.pbio.1000454 (PMC2923084; doi:10.1371/journal.pbio.1000454)
Supplement: Text S1 — We provide additional details on methods and data analysis. (0.06 MB DOC) [file pbio.1000454.s008.doc]

**SUPPORTING INFORMATION**

Figure S1 shows the presence of substantial sexual fitness variation in Riske Creek. Pollen collections from trees were completed within days of each other (April 17–22 2003). Nevertheless, viability and count can be sensitive to timing of catkin emergence and anther dehiscence [1,2]. We tested to see if sexual fitness variation was explained by the degree of catkin development using an ANOVA and found no effect (Riske Creek: F_2,94_ =2.243, P=0.112, n=96, Figure S2). If mutations accumulated primarily during shoot cell division, then variation in ramet age might explain variation in ramet sexual fitness. To test this possibility, we determined ramet age by counting rings on increment cores taken from the ramets whose pollen viability had been estimated (N = 95; two trees were not cored). Ramet age did not explain a significant amount of variation in ramet sexual fitness (Figure S3). We interpret the lack of a relationship to indicate that the number of mutations that occur within a ramet are negligible relative to the number that occur during root growth leading to each ramet.

**The role of inbreeding depression in explaining the variation in sexual fitness*.*** Theoretical work has suggested that inbreeding depression is a major factor explaining the predominance of outcrossing among mating system in trees. In particular, inbreeding depression acting across the lifespan of a plant could eliminate inbred individuals from a population [3]. Furthermore, empirical work has shown that early acting inbreeding depression is quite high in trees [4], suggesting that the level of inbreeding might be lower among those clones that survive for longer periods of time. This could potentially explain the variation in sexual fitness if short-lived inbred clones have a higher sexual fitness (e.g., if inbred individuals invest more in flowers), but then we would expect to see a positive relationship between the inbreeding level of a clone and its sexual fitness. We measured inbreeding level for the clone as the proportion of homozygous loci. As shown in Figure S4, we found no relationship between clone age and the proportion of homozygous loci at 14 different microsatellites; similarly, the proportion of homozygous loci did not explain significant variation in sexual fitness.

**Principal Component Analysis**

We first performed a PCA using a correlation matrix involving the 11 morphological traits (ramet data averaged within a clone), finding that four axes whose eigenvalues were greater than one captured the majority of the variation in the original data (79.8% of the variation). From the loadings of the variables along each axis, we assigned factor names to these four principle component (PC) axes: PC_D_1: tree size and pathogen load (pathload), PC_D_2: response to pathogen stress (response), PC_D_3: number of galls (ngalls), and PC_D_4: mechanical damage (mechdamage). As is recommended [5], product moment correlations >|0.50| were used to decide which variables had high loadings on the PC axes.

The second principal component analysis used a correlation matrix among 67 site variables, where 41 variables measured the per cent of specific plants in the understory of each patch and the other 26 were soil characteristics obtained from soil pits. In this analysis, eight axes with eigenvalues greater than one captured the majority of the variation in the original data (71.7%). A sample of factors assigned to different PC axes include: PC_E_1 (high productivity), PC_E_2 (Moisture gradient), PC_E_3 (amount of understory litter). All principal component analyses were conducted using SPSS [6]. Consultation with various site identification guides and plant identification books provided information on the environment and habitats of the different understory plants [7-10]. This was then used to determine what factor names to assign to the principal component axes.

**Multiple regression analysis of the variation in sexual fitness*.***

In the following statistical analyses, we used the lower bound estimate of the microsatellite somatic mutation rate, based on the retreat of the glaciers 10,000 years ago, to calculate clone ages. Scaling the mutation rate instead on the minimum clone age based on tree-ring data, , would not affect the significance of the results, but it would cause clone ages to be larger by a factor of 27.6.

We tested four key assumptions of multiple regression analysis in order to validate the full model as the best linear unbiased estimator of the relationship between clone age and sexual fitness. The plots of the residuals against each of the predictors revealed no departures from homoscedasticity and no evidence of serial correlation. We found no multi-collinearity among the predictors, suggesting that the regression coefficients corresponding to each predictor reflect an inherent effect of that predictor on the response variable [11]. Clone age was not significantly correlated with PC_E_2 (moisture gradient) (*r* = 0.26, t = –1.16, df = 18, P = 0.259) or with PC_D_4 (mechanical damage) (*r* = 0.29, t = 1.32, df = 18, P = 0.20). Similarly, PC_E_2 (moisture gradient) showed no significant correlation with PC_D_4 (mechanical damage) (*r* = 0.045, t = 0.19, df = 18, P = 0.85). Finally, a Shapiro–Wilk’s test showed that we could not reject the assumption of normality for the errors of the multiple regression analysis (W=0.95, P=0.38).

To confirm the association between sexual fitness and clone age, we performed three different randomization tests. In the first test, we randomly assigned sexual fitness, with replacement, to a given clone age, ignoring moisture levels and mechanical damage and performing a simple linear regression. The second test involved randomly assigning sexual fitness, moisture, and mechanical damage independently with replacement to a given clone age. In the third randomization test, we kept the relationships between clone age, moisture levels, and mechanical damage and randomly assigned sexual fitness with replacement. In each case, 10,000 randomizations were performed.

The results of our first randomization test showed that the observed correlation (calculated as Spearman’s rank rho correlation) of *r_s_* = -0.49 occurred only 2.5% of the time when clone age and sexual fitness were independent of each other (SFigure 5). Results obtained from the second and third randomization test were qualitatively the same, thus we only report results from the second. Values of the observed adjR^2^= 0.50 (from multiple regression analysis) or greater were obtained among the randomized data sets only 0.1% of the time, while a partial regression coefficient value less than or equal to that observed for clone age, -5.8
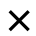
10^–5^, arose 0.13% of the time by chance.

1. Fernando DD, Cass D (1997) Developmental assessment of sexual reproduction in *Butomus umbellatus* (Butomaceae): male reproductive component. Ann Bot 80: 449-456.

2. Rajora OP, Zsuffa L (1986) Pollen viability of some *Populus* species as indicated by invitro pollen germination and tetrazolium chloride staining. Canadian Journal of Botany-Revue Canadienne De Botanique 64: 1086-1088.

3. Morgan M, Schoen D, Bataillon T (1997) The evolution of self-fertilization in perennials. The American Naturalist 150: 618-638.

4. Sorensen FC (1999) Relationship between self-fertility, allocation of growth, and inbreeding depression in three coniferous species. Evolution 53: 417-425.

5. Dillon WR, Goldstein M (1984) Multivariate Analysis: Methods and Applications. New York: John Wiley & Sons, Inc.

6. (2001) SPSS for Windows. Rel 1101

7. Antos J, Coupe, R., Douglas, G., Evans, R., Goward, T., Ignace, M., Lloyd, D., Parish, R., Pojar, R. and Roberts, A. (1996) Plants of southern interior British Columbia. Vancouver, B.C.: Lone Pine Publishing.

8. Green RN, Klinka K (1994) A field guide to site identification and interpretation for the Vancouver Forest Region. Victoria, B.C.: Research Branch of the Ministry of Forests.

9. Johnson J, Kershaw, L.J., and MacKinnon, A (1995) Plants of the Western Boreal Forest and Aspen Parkland. Edmonton, Alberta: Lone Pine Publishing.

10. Shaw RJ, On D (1979) Plants of Waterton-Glacier National Parks. Missoula, MT: Mountain Press Publishing Company.

11. Neter J, Kutner, M.H., Nachtsheim, C.J. and Wasserman, W. (1996) Applied linear statistical models. Boston: The McGraw-Hill Companies, Inc.
